# Large Language Model Few-Shot Learning for Predicting Individual Treatment Response to Smartphone-Based Mindfulness in Autistic Adults With Anxiety: Secondary Analysis of a Randomized Controlled Trial

**DOI:** 10.2196/89054

**Published:** 2026-07-23

**Authors:** Gun Ahn, Cindy E Li, Aixin Liang, Wonchang Choi, Seoin Ahn, Clark Roberts, John D E Gabrieli

**Affiliations:** 1 Department of Brain and Cognitive Science, Massachusetts Institute of Technology, 43 Vassar Street, Cambridge, MA, United States, 1 617-253-8946; 2 Hock E. Tan and K. Lisa Yang Center for Autism Research, Massachusetts Institute of Technology, Cambridge, MA, United States; 3 McGovern Institute for Brain Research, Massachusetts Institute of Technology, Cambridge, MA, United States; 4 McLean Hospital, Belmont, MA, United States

**Keywords:** machine learning, precision psychiatry, mindfulness, autism spectrum disorder, anxiety, large language model, few-shot learning, artificial intelligence

## Abstract

**Background:**

Anxiety disorders are highly prevalent among adults with autism, with 20%‐65% experiencing at least one diagnosable anxiety disorder. While mindfulness-based interventions have demonstrated efficacy for anxiety reduction, treatment response varies considerably across individuals. Machine learning approaches offer potential for identifying who is most likely to benefit from smartphone-based mindfulness interventions, enabling personalized treatment recommendations.

**Objective:**

This study aimed to develop and evaluate machine learning models to predict individual treatment response to a smartphone-based mindfulness intervention for adults with autism. We identified baseline characteristics that distinguish responders from nonresponders and explored few-shot learning with large language models (LLMs) as a complementary approach for low-data clinical prediction.

**Methods:**

We conducted a secondary analysis of a randomized controlled trial comparing a 6-week smartphone-based mindfulness intervention with a waitlist control group in adults with autism. Among 73 participants who completed the intervention, we defined responders as those achieving a ≥7-point reduction in State-Trait Anxiety Inventory state anxiety scores. Baseline predictors included demographic variables; autism trait measures; and self-report questionnaires assessing anxiety symptoms, perceived stress, affect, and mindfulness. To determine which machine learning model was most predictive of response, we trained 6 different models (logistic regression, random forest, extreme gradient boosting [XGBoost], tabular data network [TabNet], TabICL, and Tabular Prior-Data Fitted Network [TabPFN]) using nested 10-fold cross-validation with inner 5-fold cross-validation for hyperparameter tuning and evaluated GPT-4o few-shot learning with tokenized features at 20 to 70 shots.

**Results:**

Random forest achieved the highest predictive performance for state anxiety response (area under the curve [AUC] 0.79, 95% CI 0.66‐0.91), followed by TabPFN (AUC 0.78, 95% CI 0.64‐0.94) and logistic regression (AUC 0.77, 95% CI 0.73‐0.81). Higher baseline state anxiety (standardized β coefficient=1.20, *P*<.001) predicted better treatment response, while higher Autism Spectrum Quotient at baseline (standardized β coefficient=−0.17, *P*=.001), older age (standardized β coefficient=−0.18, *P*=.02), and lower childhood pretend play scores (standardized β coefficient=−0.93, *P*=.007) were associated with poorer response. Few-shot learning with 7-feature tokenization achieved an accuracy of 0.867 at 70 shots, compared to an accuracy of 0.733 for random forest. Prediction of trait anxiety changes was substantially weaker (AUCs 0.46‐0.68), likely reflecting the inherent stability of this personality dimension.

**Conclusions:**

Machine learning models successfully identified baseline characteristics predicting state anxiety response to a smartphone-based mindfulness intervention in adults with autism. Few-shot learning with LLMs demonstrated superior performance to traditional machine learning when provided with compact, high-signal feature representations, offering a promising approach for clinical prediction in small-sample settings. These findings demonstrate the feasibility of precision psychiatry in digital mental health interventions for adults with autism. As online mental health interventions become ubiquitous, patients and clinicians can know whether a particular intervention is more or less likely to benefit an individual patient.

## Introduction

### Anxiety in Adults With Autism and Mindfulness-Based Interventions

Anxiety is a prevalent and significant concern for adults with autism. Approximately 20%‐65% of adults with autism meet the diagnostic criteria for at least one anxiety disorder [1]. This finding underscores the need for effective interventions to manage anxiety symptoms in this population. Mindfulness-based interventions (MBIs) have gained interest as a potential supportive treatment because they have shown benefits for mental health, including anxiety [2]. Indeed, in our previous randomized controlled trial (RCT), a smartphone-based digital MBI produced moderate to large reductions in anxiety in adults with autism spectrum disorder [3]. Furthermore, in populations with clinical anxiety disorders, MBIs have been reported to reduce anxiety symptoms comparably to first-line treatments such as cognitive behavioral therapy [4,5].

### Need for Personalized Prediction in Digital Mental Health

Individual treatment responses and engagement patterns show considerable heterogeneity [6]. A scoping review concluded that one-size-fits-all online mindfulness interventions may be less engaging and effective than programs tailored to individual needs and preferences [7]. This variability in treatment response aligns with broader findings in mental health care: there is substantial heterogeneity in how patients with a shared diagnosis respond to the same treatment, with significant subsets showing minimal improvement [8]. Such heterogeneity has important clinical implications. When individuals experience unsuccessful treatments, they become less likely to pursue additional interventions, potentially abandoning therapeutic options from which they could benefit [9]. Personalizing treatment selection can therefore improve both efficiency—reducing unnecessary time and monetary costs—and treatment retention, by directing individuals toward interventions they are more likely to find helpful [10,11].

These considerations are especially pressing in the context of digital mental health care. Smartphone-based mindfulness programs are now widely accessible and increasingly promoted to adults with autism, yet almost no evidence exists to inform whether a given individual is more or less likely to benefit from such an intervention. Patients and clinicians currently have no empirical basis for deciding whether to invest the time and effort a digital mindfulness program requires—and without such guidance, treatment decisions default to trial and error. Predicting individual clinical response to online mindfulness interventions is therefore the central aim of this study. Such predictions could provide patients with actionable, evidence-based guidance before committing to a course of treatment.

### Machine Learning and Few-Shot Learning for Low-Data Clinical Prediction

Machine learning can enhance precision in mental health interventions through pattern recognition in high-dimensional baseline data, capturing complex relationships that traditional statistical methods may miss. By leveraging high-dimensional data, machine learning models can potentially detect complex patterns—for example, combinations of baseline characteristics—that predict treatment response on an individual level. Applications of machine learning in psychiatry have shown encouraging accuracy in classifying responders versus nonresponders to treatment. A 2025 meta-analysis of 155 studies reported that machine learning algorithms achieved a mean area under the curve (AUC) of approximately 0.80 in predicting treatment outcomes for anxiety and depression, indicating good discrimination between responders and nonresponders on average [12]. Crucially, studies that used rigorous validation methods (eg, avoiding overfitting by using proper cross-validation) tended to report higher predictive accuracy. These findings establish that machine learning models trained on baseline clinical characteristics can reliably identify who is likely to respond to treatment—a capacity that, if applied to adults with autism receiving mindfulness interventions, could identify which individuals are most likely to benefit from a mindfulness app and who might need alternative or augmented support. However, a central challenge in applying machine learning in this context is sample size: RCTs conducted in specialized clinical populations such as adults with autism necessarily yield small datasets, and traditional supervised learning models are known to underperform and overfit under such low-data conditions—precisely the setting most in need of personalized prediction.

Clinical prediction faces a fundamental tension: rare conditions, narrow subgroups, and underrepresented communities most requiring personalized approaches typically yield the smallest training datasets [13]. This tension is directly relevant to the present study, where the available sample from our RCT is limited. Identifying reliable baseline predictors of treatment response is the core aim of this work, yet this aim is most at risk of being undermined by insufficient sample size for traditional supervised classifiers. Addressing this methodological constraint is therefore not a secondary objective but a prerequisite for achieving the study’s primary goal. Large language models (LLMs) offer a potential solution through few-shot learning, where foundation models leverage extensive pretraining to perform classification tasks with only a handful of demonstrations [14]. Research demonstrated that serializing tabular clinical data into natural language strings enables effective few-shot classification, with performance competitive to traditional machine learning in low-sample regimes [15]. Given these properties, LLMs represent a methodologically motivated response to the small-sample challenge inherent in this study rather than a separate line of inquiry. Therefore, we additionally explored whether LLMs—without gradient-based fine-tuning—could classify future treatment responders from tokenized baseline data using only few-shot examples and under what conditions these models approximate or exceed the performance of traditional supervised classifiers.

### Study Aims

This study pursued two complementary aims. The first aim was to identify baseline predictors of anxiety reduction in adults with autism following a smartphone-delivered mindfulness intervention. This study is a secondary analysis of data from a previously published RCT (ClinicalTrials.gov NCT05880498), which demonstrated that a 6-week self-guided mindfulness intervention using the Healthy Minds Program smartphone app significantly reduced anxiety symptoms and perceived stress in adults with autism compared to a waitlist control (with medium to large effect sizes) [3]. These findings were largely replicated when the waitlist control group used the app, and the benefits were largely retained after a 6-week delay.

The second aim was to examine whether baseline characteristics—demographics, autism trait measures, and self-report questionnaire scores on anxiety symptoms, perceived stress, affect, and trait mindfulness—could predict clinically meaningful anxiety reduction. We applied a range of machine learning models to the baseline data to develop predictive classifiers of treatment response. Given the inherent sample size limitations of this specialized RCT, we also evaluated whether LLM-based few-shot learning could identify these predictive patterns more effectively than traditional supervised methods—directly serving the study’s core aim of reliable, individualized prediction. By leveraging ML, we aimed to move beyond group-level effects and toward precision insights, ultimately to inform more tailored mental health interventions for the autistic community.

## Methods

### Study Design and Participants

#### Description of the Original RCT’s Design

Participants completed the entire study remotely. Eligible individuals were administered baseline assessments and then randomly assigned to either the intervention group or control group through block randomization, a pseudorandomization process designed to address potential baseline differences between groups for the main outcome measure (anxiety). We pseudorandomly assigned participants in blocks based on their low, medium, or high baseline trait anxiety scores to ensure similar representation of trait anxiety scores between groups. The cutoffs for the trait anxiety scores from the State-Trait Anxiety Inventory (STAI) for Adults were based on prior studies that typically consider STAI scores to be high at 45 and above [16,17] and have categorized scores into “no or low anxiety” (20-37), “moderate anxiety” (38-44), and “high anxiety” (45-80) [18]. Neither researchers nor participants were blinded to group assignment.

#### Participant Recruitment

Individuals were recruited from January through November 2022 from two databases: (1) the Autism Research Participant Database at the Massachusetts Institute of Technology, which consists of over 150 adults recruited from the community with an existing clinical diagnosis of autism and who met the Autism Diagnostic Observation Schedule criteria [19], and (2) Simons Foundation Powering Autism Research for Knowledge (SPARK) database, which consists of over 20,000 adults with a professional autism diagnosis [20] and is confirmed to have a high degree of autism diagnosis validity [21]. Details are reported elsewhere [3].

#### Demographic Characteristics

Of 173 adults with autism who were invited to participate in the study, 117 completed the screening measures. Of those, 91 individuals met the eligibility criteria, and 89 completed the baseline assessments and were randomly assigned to either the intervention group (n=45) or the waitlist control group (n=44). However, the data of only 73 individuals were used for this study. Participants who completed the baseline evaluations and check-ins but not the posttest were omitted to ensure the robustness of the data. Participants who did not sufficiently participate in the program and those with a large number of missing data points were also omitted to maintain the quality of analysis. Of the 73 individuals, 37 belonged to the intervention group and 36 belonged to the waitlist control group. The intervention lasted for 6 weeks.

### Intervention

The intervention involved a customized version of the Healthy Minds Program smartphone app delivered over 6 weeks. Full details of the curriculum and app customization are described elsewhere [3].

### Measures

All measures are described in detail elsewhere [3]. Briefly, baseline assessments included demographic information, autism trait measures (Social Responsiveness Scale, Second Edition [SRS-2] [22]; Autism Spectrum Quotient [AQ] [23]; and Comprehensive Autistic Trait Inventory [CATI] [24]), anxiety symptoms (STAI [25] and Patient-Reported Outcomes Measurement Information System [PROMIS] Anxiety [26]), perceived stress (Perceived Stress Scale [PSS] [27]), positive and negative affect (Positive and Negative Affect Schedule [PANAS] [28]), and trait mindfulness (Mindful Attention Awareness Scale [MAAS] [29] and Five Facet Mindfulness Questionnaire-15 [FFMQ-15] [30]).

### Outcome Variable

We deemed participants with an improved score of 7 points or more on the STAI as “responders,” meaning that they reported meaningful change in their STAI anxiety score. This was determined based on the following factors. First, as mentioned above, we used the guidelines provided by Kayikcioglu et al [18] who categorized scores into “no or low anxiety” (20-37), “moderate anxiety” (38-44), and “high anxiety” (45-80). Given that the moderate anxiety category consists of a 6-point range, we considered greater than 6 points to represent meaningful change, as this is the smallest change that would move an individual from one category to another. Second, other studies have defined clinically meaningful change on the STAI as a decrease of 6 points or more [31] and 8 points or more [32].

### Data Preprocessing and Feature Engineering

Prior to model building, we performed several data preprocessing steps to clean and encode the baseline data for analysis:

1. Data cleaning and coding: we standardized variable names and ensured that all questionnaire items were coded in the same direction. For instruments with reverse-scored items (eg, “I feel calm” on the STAI is reverse scored for anxiety), we inverted the scores so that higher values uniformly indicated greater symptom severity or trait level. Likewise, categorical response options (eg, Likert scales from “Never” to “Always”) were mapped to numeric values in a consistent manner across scales. Missing data were minimal due to the online surveys requiring answers for all items.
2. One-hot encoding of categorical variables: categorical demographic variables such as sex and race were encoded as binary dummy variables (eg, Female=1, Male=0; White=1, Non-White=0) so that they could be included in the models. This avoids any ordinal assumptions about these categories.
3. Normalization of continuous features: continuous numeric features (eg, age) and questionnaire scale scores were examined for distribution. In general, we rescaled features to a 0 to 1 range or *z* score–standardized them to mean 0 (SD 1). Importantly, all scaling and standardization procedures were applied after splitting data into training and test folds, using parameters learned from the training set only, to prevent any information leakage. We implemented these transformations within a pipeline that ensured that the test data was never used to fit preprocessing parameters.
4. Train-test splitting: although the primary analysis used cross-validation (described below), we maintained a separation between model development and final evaluation. All preprocessing steps were encapsulated and repeated within each cross-validation fold such that the model training on each fold was blind to the held-out data.

Given that the primary objective of this study was to identify specific factors influencing the effectiveness of the mindfulness intervention, rather than merely maximizing predictive accuracy, our feature selection process prioritized clinical interpretability. We carefully selected and preprocessed baseline characteristics to prioritize clinical interpretability to ensure that the subsequent models could capture meaningful, actionable patterns relevant to personalized treatment response.

### Machine Learning Models and Training Procedure

#### Predictive Models

We developed predictive models to classify participants as responders or nonresponders using their baseline features. A diverse set of machine learning algorithms was used, ranging from simple linear models to advanced neural network approaches for tabular data. The following models were trained and evaluated:

- Logistic regression: this is a generalized linear model that predicts the log odds of response as a weighted sum of the input features. We used L1-regularized logistic regression as a baseline. This model assumes linear relationships between predictors and the outcome and is easily interpretable via its coefficients.
- Random forest: this is an ensemble of decision trees that votes on the outcome class. Random forests handle nonlinear feature interactions and are relatively robust to overfitting [33].
- Extreme gradient boosting (XGBoost): this is a boosted tree model that sequentially builds an ensemble of trees, where each new tree corrects the errors of the previous ones. XGBoost often achieves high performance on tabular data by optimizing a specified loss with regularization. We tuned the key hyperparameters such as the learning rate, maximum tree depth, and L1/L2 regularization weights [34].
- Tabular data network (TabNet): this is a deep learning model specifically designed for tabular data [35]. TabNet uses an interpretable neural architecture with sequential attention to select which features to attend to at each decision step. This allows the model to learn feature importance in a sparse, instance-wise manner.
- TabICL: this is an in-context learning approach for tabular data [36]. In line with advances in transformer models for small tabular datasets, we experimented with a transformer that treats each data point’s feature set as a sequence and was pretrained to internally simulate algorithmic reasoning. This experimental model is based on the idea of prior-data fitted networks; however, given our data size, we primarily report results from more established models.
- Tabular Prior-Data Fitted Network (TabPFN): this is a transformer-based “tabular foundation model” that is pretrained on millions of synthetic datasets to solve tabular classification tasks without extensive training on the new data [37]. TabPFN leverages in-context learning and Bayesian principles to make rapid predictions on small datasets. We used the open-source implementation from the authors and applied it to our data. Notably, TabPFN requires no hyperparameter tuning and outputs class probabilities in a single forward pass, making it an attractive option for small N problems.

To ensure fair comparison, we trained each model within a nested cross-validation framework (described below) and evaluated them primarily on their ability to discriminate responders from nonresponders.

#### Training and Validation Strategy

We used nested cross-validation to optimize model hyperparameters while providing an unbiased estimate of performance. In the outer loop, we performed a stratified k-fold split of the 73 data points (k=10 folds), ensuring that each fold had a similar responder versus nonresponder ratio. For each outer fold, an inner loop of cross-validation was used on the training portion to select the best hyperparameters (eg, regularization strength for logistic regression, tree depth and number for random forest, learning rate and tree complexity for XGBoost, etc).

The outer loop generates 10 folds, where one-tenth of the data in each fold is labeled as the test set, the holdout data for this outer fold. The held-out tenth of the data in each fold differs, and the model would not see during any part of this holdout data in its training or hyperparameter tuning. The remaining 90% is then used in inner cross-validation (k=5 folds) for hyperparameter search, combined with grid search or random search over a predefined parameter grid for each model. That 90% of data is split into 5 folds, where 80% of each fold is used to train the model with the different combinations of hyperparameters, and 20% is the validation set. The combination of hyperparameters that yielded the best result from the validation set, averaged across all 5 inner folds, is stored and then tested on the holdout data from the outer loop. In short, for each of the 10 outer folds, there are 5 inner folds to find the most optimal parameters. The best set of parameters resulting from the inner cross-validation is used to construct a model to evaluate the holdout set from the respective outer fold, providing an AUC value. After performing nested cross-validation, there are 10 different models, where each has the best set of parameters from its respective inner cross-validation. These 10 models are then retrained on the entire dataset with their respective parameters and evaluated using stratified 5-fold cross-validation. The resulting output is the mean AUC averaged across 5 held-out test sets and CIs for each of the 10 models. The model with the best CI and/or mean AUC is chosen as the final model for further evaluation (feature importances, SHAP analysis, etc). Nested cross-validation protects against overfitting during model tuning and provides a more rigorous assessment of how the models would perform on new data [38]. We used area under the receiver operating characteristic curve (AUC-ROC) as the primary performance metric since it is threshold independent and suitable for binary classification with potentially imbalanced classes. CIs for AUC were obtained by treating the AUC values from the 5 cross-validation folds as independent estimates and computing the mean and 95% CI across these folds.

### Feature Importance and Interpretation

#### Shapley Additive Explanations

To provide a unified and model-agnostic interpretation across different algorithms, we used Shapley Additive Explanations (SHAP). Based on the cooperative game theory, SHAP values attribute the prediction of each instance to its features by quantifying their marginal contributions. Specifically, a feature’s marginal contribution is calculated as the difference in prediction between a subset of features that includes it and one that does not. The final SHAP value is derived as the weighted average of these marginal contributions across all possible coalitions, ensuring a fair distribution of the model’s total predictive power [39].

Crucially, the deployment of architecturally diverse models was not merely for performance comparison but was integral to answering our primary research question regarding the nature of treatment predictors. Since clinical outcomes in autism are likely driven by complex, nonlinear interactions rather than simple linear associations, relying on a single modeling approach could miss critical prognostic factors. In the context of this study, SHAP serves 3 critical interpretability functions to synthesize findings across these diverse algorithms.

- Model-agnostic validation: by computing SHAP values across architecturally diverse models (tree-based ensembles and deep tabular networks), we leverage its unified framework to distinguish true clinical signals—features that consistently drive predictions regardless of specific modeling assumptions—from algorithm-specific artifacts.
- Local explainability: SHAP’s local explainability allows us to trace why a particular participant was predicted as a responder or nonresponder, revealing not just which baseline characteristics matter but also how their specific values (eg, a state anxiety score of 55 vs 40) shift predicted outcomes in a clinically interpretable direction.
- Consistent global ranking: unlike traditional feature importance metrics (eg, Gini impurity for random forest vs information gain for XGBoost), which are model specific and often incomparable, SHAP provides a unified measure of global feature importance.

This allowed us to rigorously identify the most influential predictors across the entire cohort by aggregating the absolute SHAP values, ensuring that our key findings were robust across different algorithmic approaches. Collectively, this interpretability framework ensured that our machine learning analysis yielded actionable insights for personalized treatment decision-making rather than merely optimizing predictive metrics.

#### Interpretation Methods

To interpret which baseline features were most predictive of treatment response, we examined model-specific indicators of feature importance, organized by model complexity. For logistic regression, we inspected standardized beta coefficients and their statistical significance (*P* values) for each predictor. For tree-based models such as random forest and XGBoost, we computed feature importance scores derived from Gini impurity reduction (random forest) or information gain (XGBoost), averaged across trees and cross-validation folds. We also used SHAP analyses to quantify each feature’s contribution to individual predictions for random forest, XGBoost, TabICL, and TabPFN. In addition, TabNet’s inherent attentive feature selection masks were aggregated to identify the most frequently attended features across samples. To minimize the risk of overinterpretation, we focused on features that consistently showed high importance across multiple folds and models.

### Few-Shot Learning With LLMs

To evaluate whether LLMs could predict treatment response with minimal training examples, we implemented a few-shot learning approach using GPT-4o models accessed via the OpenAI API. This approach complemented our traditional machine learning analyses by testing whether foundation models’ pretrained knowledge can support clinical prediction in low-data settings. The full prompting procedure used for LLM classification, including exact prompts and parsing rules, is provided in the Multimedia Appendix 1 for reproducibility.

#### Feature Selection for Tokenization

Based on feature importance analyses from the supervised models described above, we selected a compact set of 7 features for the primary few-shot experiments: five individual STAI state anxiety items (17, 13, 1, 4, and 7—items assessing the feelings of worry, nervousness, calmness [reversed], tension, and anticipatory concern, respectively) showing the strongest associations with treatment response, AQ item 40 (childhood pretend play enjoyment), and age. Critically, this same 7-feature set was used as the input for both the LLM few-shot classifier and the random forest baseline reported in this comparison. The LLM did not receive any additional features, and random forest was retrained on this identical 7-feature input—not the full feature set—for this specific comparison. This ensures that any performance difference between the two approaches reflects differences in the learning mechanism (few-shot in-context learning vs supervised training) rather than differences in the information available to each model. The purpose of this feature set was not to build an independent predictive model but to evaluate whether LLM can make use of a small number of clinically interpretable variables which is known to relate to treatment response. The 7 features were determined once and kept fixed for all experiments and not tuned based on LLM performance.

#### Tokenization Strategy

Features were serialized into comma-separated sequences following a fixed order based on feature importance (eg, “STAI_17=0.67, STAI_1=0.67, STAI_4=0.33, STAI_13=0.67, STAI_7=1.0, AQ40=1.0, Age=40.0”). All continuous variables were normalized to the 0 to 1 range. The token sequence was paired with an instruction prompt defining the classification task and the response criterion of ≥7-point reduction in STAI scores. To examine how feature dimensionality influences performance, we additionally tested comprehensive tokenization conditions using the top 50 and 100 features ranked by random forest importance.

#### Few-Shot Configuration

We used 5-fold stratified cross-validation matching our supervised learning approach. The temperature parameter was fixed at 0 for all experiments. For each fold, we randomly sampled k labeled training examples (k ∈ {20, 30, 40, 50, 60, 70}) with stratification to preserve the empirical responder rate (~40%) within the prompt. The model was instructed to output a binary prediction in structured JSON format. If the output did not contain a valid label after JSON and pattern extraction, the prediction was assigned the label 0. The LLM was evaluated on the same held-out test set used for the random forest baseline. Mean classification accuracy was computed over cross-validation folds and repetitions, with 95% CIs derived using a t-distribution. All analytical procedures were conducted in Python (version 3.12; Python Software Foundation). The study received ethics approval, and all participants provided informed consent in the original RCT. For this secondary analysis, we used deidentified data.

### Ethical Considerations

The original study was approved by the Massachusetts Institute of Technology Committee on the Use of Humans as Experimental Subjects (Institutional Review Board COUHES protocol 2105000376). All procedures in the study were performed in accordance with the ethical standards of the 1964 Helsinki Declaration and its later amendments or comparable ethical standards. Informed written consent was obtained from all individuals before participation in the original RCT, as reported previously [3]. For this secondary analysis, we used deidentified data.

## Results

### Model Performance

Across the 73 participants, approximately half met the responder criterion of ≥7-point state anxiety reduction post intervention (32/73, 43.8% of the intervention group were responders, by definition). We first report on the ability of various machine learning models to classify these responders versus nonresponders using only baseline data. Overall, models achieved statistically significant prediction of STAI state response (AUC values 0.67‐0.79, all 95% CIs excluding 0.5 across the cross-validation folds). In contrast, when predicting change in trait anxiety, AUCs ranged from ~0.46 to 0.68.

**Figure 1.**
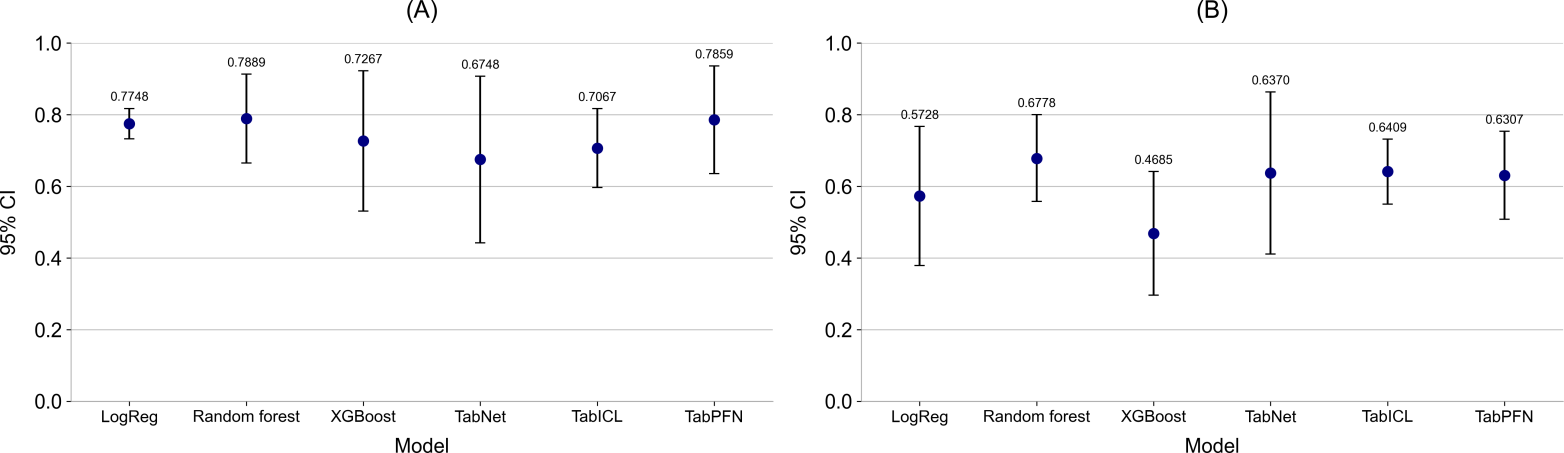
Comparison of AUC scores for 6 models—logistic regression, random forest, XGBoost, TabNet, TabICL, and TabPFN—using the State-Trait Anxiety Inventory (STAI) dataset. The figure reports the CIs of the AUC for each model evaluated on (A) STAI state and (B) STAI trait data. Ensemble tree-based methods showed the highest performance among all models tested. AUC: area under the curve; LogReg: logistic regression; TabNet: tabular data network; TabPFN: Tabular Prior-Data Fitted Network; XGBoost: extreme gradient boosting.

Figure 1 shows that among the models tested, the ensemble tree–based methods performed best. The random forest classifier achieved the highest AUCs on average for both outcomes. For STAI state response, the random forest AUC was approximately 0.79 (95% CI 0.66‐0.91), making it the top-performing model. The TabPFN model was a close second, with an AUC of approximately 0.79 (95% CI 0.64‐0.94), followed by logistic regression, with an AUC of around 0.78 (95% CI 0.73‐0.81).

TabNet did not exceed the tree models, possibly reflecting the challenge of tuning a complex neural model on a relatively small dataset (n=73). Similarly, the TabICL approach (transformer with in-context learning) did not outperform the more established methods.

### Feature Importance

We examined feature importance scores, SHAP values, and model coefficients to infer the top predictors of being a responder. Despite the different methodologies of our models, there was notable convergence on several key variables related to baseline anxiety levels and autism characteristics. Feature importance values from all models are presented in Figures S1 and S2 in Multimedia Appendix 2.

#### Baseline Anxiety as the Primary Predictor

**Figure 2.**
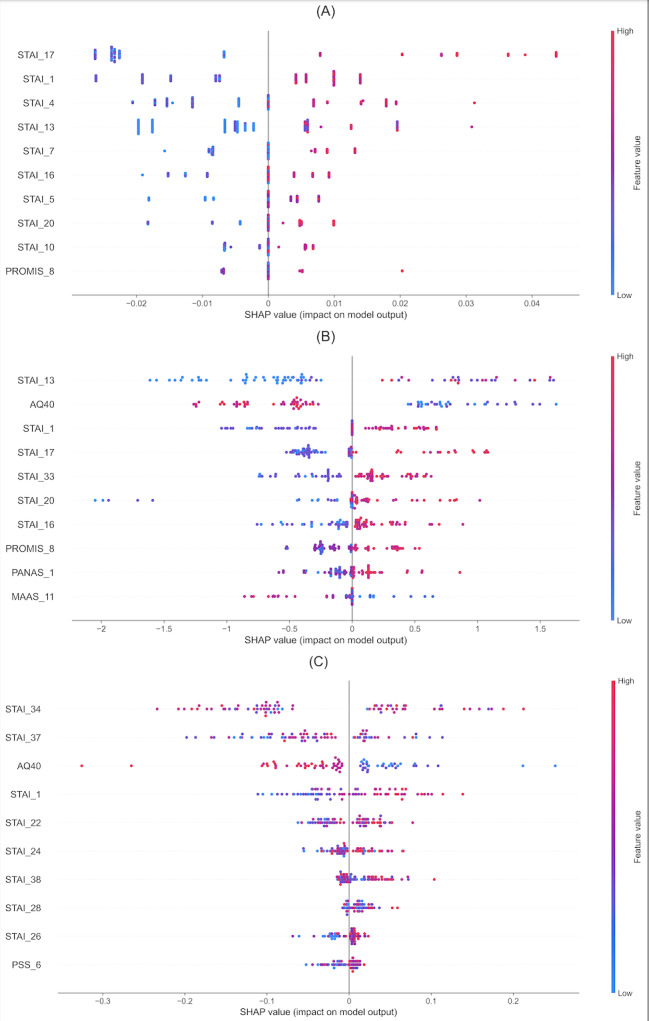
The impact of individual features on model output, with color indicating the feature value and horizontal position representing the SHAP value: (A) random forest, (B) XGBoost, and (C) TabPFN. The comparison highlights model-specific differences in feature importance attribution. Features are vertically ordered according to their mean absolute SHAP values, with the most influential features appearing at the top of each plot, facilitating consistent comparison of feature importance across models. AQ: Autism Spectrum Quotient; MAAS: Mindful Attention Awareness Scale; PANAS: Positive and Negative Affect Schedule; PROMIS: Patient-Reported Outcomes Measurement Information System; PSS: Perceived Stress Scale; SHAP: Shapley Additive Explanations; STAI: State-Trait Anxiety Inventory; TabPFN: Tabular Prior-Data Fitted Network; XGBoost: extreme gradient boosting.

Participants with higher initial state anxiety were substantially more likely to experience significant anxiety reduction from the intervention. In the logistic regression model (Table 1), baseline STAI state anxiety was the strongest predictor (standardized β coefficient=1.20, *t*_71_=14.12, *P*<.001); each 1-point increase in baseline state anxiety was associated with 3.32 times higher odds of treatment response. This pattern was corroborated by SHAP analyses across all 3 top-performing models, where the baseline STAI state anxiety items dominated the most influential features: 5 of the top 5 features in random forest (Figure 2A), 4 of the top 5 in XGBoost (Figure 2B), and 4 of the top 5 in TabPFN (Figure 2C) were specific items from the STAI questionnaire reflecting acute anxiety symptoms.

**Table 1.** Logistic regression coefficients for predicting treatment response.

Feature	β^a^	*t* test (*df*)	*P* value
STAI^b^ baseline state anxiety	1.20	14.12 (71)	<.001
AQ^c^ baseline	−.17	−8.54 (71)	.001
Age	−.18	−3.70 (71)	.02
AQ40: pretend play enjoyment^d^	−.93	−5 (71)	.007

a β: standardized regression coefficient.

b STAI: State-Trait Anxiety Inventory.

c AQ: Autism Spectrum Quotient.

d AQ40 refers to the Autism Spectrum Quotient item “When I was young, I used to enjoy playing games involving pretending with other children.”

Feature importance and SHAP values from random forest were the baseline rating on the STAI state anxiety item assessing worry (STAI_17; Table 2). The SHAP summary plot reveals that higher ratings on this item (indicating greater worry) consistently pushed predictions toward responder status. Similarly, items assessing calmness (reverse-scored), tension, and nervousness (all STAI state items) emerged as strong predictors, with scores endorsing less calmness, greater tension, and more nervousness at baseline associated with higher probability of response.

**Table 2.** Top 10 feature importance scores from the random forest model.

Question	Feature importance^a^
STAI_17^b^	0.989
STAI_13	0.407
STAI_1	0.400
STAI_4	0.394
STAI_7	0.249
STAI_16	0.227
STAI_5	0.188
STAI_20	0.177
PROMIS_8^c^	0.133
PSS_10^d^	0.114

a Feature importance scores indicate the relative contribution of each baseline variable to the random forest classifier's predictions.

b STAI: State-Trait Anxiety Inventory.

c PROMIS: Patient-Reported Outcomes Measurement Information System.

d PSS: Perceived Stress Scale*.*

#### Childhood Pretend Play as a Key Autism-Specific Predictor

Notably, the second most important feature in the XGBoost model (Figure 2B) and third most important feature in the TabPFN model (Figure 2C) was an item from the Autism Spectrum Quotient (AQ): “When I was young, I used to enjoy playing games involving pretending with other children (AQ40).” The logistic regression (Table 1) analysis confirmed this variable’s significance (standardized β coefficient=−0.93, *t*_71_=−5, *P*=.007). For this item, responses were coded as 0 (indicating higher imagination/enjoyment of pretend play) and 1 (indicating lower imagination/less enjoyment of pretend play). The negative coefficient indicates that lower scores on this item predicted better treatment response. The SHAP summary plot demonstrates this pattern clearly: participants with lower feature values (blue dots, indicating they enjoyed pretend play more) showed positive SHAP values, pushing predictions toward responder status. Additionally, XGBoost’s aggregate feature importance rankings (Table 3) identified another imagination-related item (AQ3: “If I try to imagine something, I find it very easy to create a picture in my mind”) as the seventh most important predictor.

**Table 3.** Aggregate feature importances over 5 k-fold (stratified) cross validation for the final selected XGBoost^a^ model.^b^

Question	Feature importance^c^
STAI_17^d^	0.165
STAI_1	0.146
STAI_20	0.139
PSS_6^e^	0.106
STAI_19	0.096
STAI_13	0.093
AQ3^f^	0.092
FFMQ_6^g^	0.086
AQ32	0.082
STAI_30	0.082

a XGBoost: extreme gradient boosting.

b The top 10 predictors for this model are displayed in order of most to least influential. Similar to the random forest model results, the State-Trait Anxiety Inventory (STAI) questions appear to be more influential predictors compared to other measures.

c Feature importance scores represent gain-based importance averaged across the 5 cross-validation folds. Higher values indicate greater contribution to the model’s predictive accuracy.

d STAI: State-Trait Anxiety Inventory.

e PSS: Perceived Stress Scale.

f AQ: Autism Spectrum Quotient.

g FFMQ: Five Facet Mindfulness Questionnaire*.*

#### Age as a Predictor

Age also emerged as a significant predictor of treatment response. The logistic regression model revealed that older age was associated with poorer outcomes (standardized β coefficient=−.18, *t*_71_=−3.70, *P*=.02), indicating that younger participants tended to respond better to the smartphone-based mindfulness intervention. Of note, age did not appear among the top features in any of the SHAP analyses for the random forest, XGBoost, or TabPFN models, suggesting its predictive contribution may be more modest compared to baseline anxiety severity.

### Few-Shot Learning Performance

Table 4 presents classification accuracy as a function of training sample size (20‐70 shots) for the random forest baseline and the LLM tokenization conditions. Random forest model performance was relatively stable across shot counts, with the accuracy ranging from 0.715 to 0.765. The 7-feature LLM tokenization began slightly below random forest at 20 to 40 shots but exceeded it from 50 shots onward. At 70 shots, the 7-feature LLM achieved an accuracy of 0.867 (95% CI 0.640‐1), higher than the random forest baseline of 0.733 (95% CI 0.387‐1).

In contrast, the 50-feature and 100-feature tokenization conditions underperformed compared to both random forest and the 7-feature condition at lower shot counts. Only at the 70-shot training size did the 100-feature condition approach baseline performance (accuracy 0.800, 95% CI 0.245‐1), while still trailing the minimal tokenization approach.

**Table 4.** Comparison of model performance (accuracy) across training sample sizes using few-shot learning (Italicized values indicate the best-performing condition at each training sample size.).

Training size	RF^a^ baseline		7-Feature token		50-Feature token		100-Feature token	
	Accuracy	95% CI	Accuracy	95% CI	Accuracy	95% CI	Accuracy	95% CI
20-shot	*0.740*	0.709‐0.770	0.725	0.682‐0.767	0.626	0.561‐0.691	0.604	0.551‐0.656
30-shot	*0.726*	0.688‐0.763	0.726	0.648‐0.803	0.614	0.531‐0.697	0.595	0.505‐0.686
40-shot	*0.715*	0.647‐0.783	0.703	0.636‐0.770	0.630	0.589‐0.671	0.612	0.515‐0.710
50-shot	0.756	0.695‐0.818	*0.765*	0.704‐0.827	0.661	0.616‐0.706	0.687	0.583‐0.791
60-shot	0.754	0.629‐0.878	*0.785*	0.589‐0.980	0.677	0.572‐0.782	0.661	0.442‐0.881
70-shot	0.733	0.387‐1	*0.867*	0.640‐1	0.733	0.387‐1	0.800	0.245‐1

a RF: random forest.

## Discussion

### Principal Findings

#### Summary of Key Findings

We applied machine learning to predict anxiety symptom reduction in adults with autism receiving a smartphone-based mindfulness intervention. Among 73 participants, random forest achieved the highest predictive performance (AUC 0.79, 95% CI 0.66‐0.91) for identifying state anxiety responders, with TabPFN (AUC 0.78) and logistic regression (AUC 0.77) showing comparable accuracy. Prediction of trait anxiety changes was substantially weaker (AUCs 0.46‐0.68). Two baseline characteristics emerged as consistent predictors across models: higher baseline state anxiety predicted better response (standardized β coefficient=1.20, *P*<.001), and less childhood enjoyment of pretend play predicted poorer response (standardized β coefficient=−0.93, *P*=.007). Older age additionally predicted poorer response in logistic regression (standardized β coefficient=−0.18, *P*=.02), although this effect was not prominent in tree-based model SHAP analyses, suggesting it warrants cautious interpretation. Few-shot learning with LLMs using 7-feature tokenization achieved 86.7% accuracy at 70 training examples, significantly outperforming random forest (73.3%). Overall, these findings indicate that machine learning models were useful predictors of individual reduction in anxiety in response to the smartphone intervention.

#### Model Performance and Clinical Utility

The predictive accuracy achieved in this study (AUC 0.79) falls within the range considered clinically meaningful for treatment selection decisions. Conventional standards in clinical medicine interpret AUC values of 0.70 and higher as indicating fair accuracy, with values of 0.80 and higher indicating optimal accuracy [40]. A systematic review and meta-analysis of externally validated models for mood, anxiety, and psychotic disorders reported a summary AUC of 0.72 for predicting treatment outcomes—primarily remission versus nonremission or response versus nonresponse—across both pharmacological and behavioral interventions [41]. The predictive accuracy (AUC 0.79) reflects realistic expectations for prediction from baseline characteristics alone, particularly in heterogeneous populations such as adults with autism. Calibration analyses revealed that both the logistic regression and random forest models showed adequate correspondence between predicted probabilities and observed response rates (Figure S3 in Multimedia Appendix 2), suggesting that these predictions are well calibrated despite the modest sample size and supporting the robustness of the findings.

Random forest and TabPFN classifiers showed optimal predictive accuracy for responder status, with random forest demonstrating slightly higher AUC and more consistent cross-validation performance. These findings suggest that machine learning models can successfully identify baseline characteristics that distinguish individuals most likely to benefit from smartphone-delivered mindfulness training for anxiety symptom reduction. The nested cross-validation approach, while computationally intensive, provided rigorous protection against overfitting during model selection. By separating hyperparameter tuning (inner cross-validation) from performance estimation (outer cross-validation), we obtained more realistic estimates of how these models would perform on truly unseen data. The relatively narrow confidence intervals for logistic regression and random forest suggest stable performance, enhancing confidence in their clinical utility.

#### State Versus Trait Anxiety Prediction

A striking finding was the marked difference in predictive performance between state and trait anxiety outcomes. The models were successful at identifying participants likely to benefit in terms of state anxiety reduction but could not confidently predict who would exhibit broader anxiety trait improvements. While our models achieved moderate success in predicting state anxiety response (AUC 0.67‐0.79), prediction of trait anxiety changes was substantially weaker (AUCs 0.46‐0.68) and often not significantly different from chance. The logistic regression model showed better calibration and less variability when predicting the state anxiety outcome than the trait anxiety outcome, with the linear model appearing to struggle when the outcome was defined by trait changes. The distinction between state and trait anxiety, as articulated by Spielberger, reflects meaningful differences in both conceptualization and temporal stability [25]. State anxiety is defined as “a transitory emotion characterized by physiological arousal and consciously perceived feelings of apprehension, dread, and tension,” whereas trait anxiety represents “an individual’s predisposition to respond” to perceived threats [42]—essentially a stable personality characteristic. Trait anxiety is conceptualized as part of negative affectivity or neuroticism, which “is hypothesized to be a relatively stable construct, and measures of negative affectivity/neuroticism have high test-retest reliability over periods of years” [43]. This fundamental stability poses a significant challenge for prediction in the context of short-term interventions. Although the 6-week mindfulness intervention produced some reductions in trait anxiety at the group level, trait anxiety was not informative for the prediction of treatment efficacy. These findings carry an important implication for how claims about “anxiety reduction” should be interpreted in this literature: meaningful predictive precision was achievable for state anxiety—a tractable, intervention-sensitive outcome—but not for trait anxiety, and future precision psychiatry studies should specify which anxiety dimension they target, both in their outcomes and in their predictive claims. Longitudinal studies may also examine if reductions in state anxiety translate over time into reductions in trait anxiety.

#### Key Predictors of Treatment Response

Across models, feature importance metrics consistently identified several primary predictors. However, because different machine learning methods make different assumptions and optimize different objectives, we treat convergence across models as stronger evidence than findings from any single model and discuss model-specific findings with appropriate caution. Higher baseline state anxiety (STAI_Baseline_STATE standardized β coefficient=1.20, *P*<.001) predicted better treatment outcomes, and this finding was the most consistent across all 3 models: STAI items occupied the majority of top-ranked features in both the random forest and XGBoost models, lending convergent validity to this predictor. Those who started out more anxious showed greater improvement, suggesting that participants with more room for improvement benefited more from the intervention. This finding is commonly observed in anxiety treatment research and aligns with the clinical intuition that those with greater symptom severity have more room for improvement. Importantly, the RCT design controls for potential regression to the mean effects, as both intervention and waitlist groups ideally should experience similar natural fluctuations in anxiety levels; thus, the observed relationship reflects genuine treatment response rather than a statistical artifact. The finding that baseline anxiety severity predicts treatment response parallels results for other digital mental health interventions; both Karyotaki et al [44] and Mohr et al [45] found that higher baseline symptom severity was associated with greater absolute improvement in internet-based and app-delivered interventions, respectively. Similarly, studies of mindfulness-based interventions in nonautistic populations have consistently found that individuals with higher baseline symptoms show larger absolute reductions; Greeson et al [46] and Arch and Ayers [47] both reported this pattern across mindfulness-based stress reduction and group-based mindfulness programs for anxiety, respectively. The present study extends these findings to adults with autism, a population that has received limited attention in precision mental health research despite high rates of anxiety disorders.

Conversely, older age (standardized β coefficient=−0.18, *P*=.02) was associated with poorer response in the logistic regression model. However, it is notable that age did not emerge as a prominent predictor in the random forest or XGBoost feature importance rankings, suggesting that this effect may be more detectable under the linear assumptions of logistic regression than under the nonlinear, interaction-sensitive frameworks of tree-based models. This discrepancy warrants caution: rather than concluding definitively that age moderates treatment response, we interpret this as a hypothesis-generating finding that warrants replication in larger samples. Younger participants tended to respond better to the smartphone-based mindfulness intervention. The age effect suggests that younger adults with autism may engage more readily with smartphone-based interventions or that more years of anxiety may render patients to be less plastic in response to this treatment. However, the modest effect size suggests that age should not be an exclusionary factor in treatment recommendations. The age-related moderation effect aligns with prior findings in the mindfulness literature. Prior research has similarly found that younger participants benefit more from in-person interventions [48].

Less childhood enjoyment of pretend play (standardized β coefficient=−0.93, *P*=.007) was associated with poorer response. This predictor showed notable consistency across models: it was the second-ranked feature in XGBoost and third-ranked feature in TabPFN by SHAP value, in addition to being a significant predictor in logistic regression. This convergence across 3 methodologically distinct models—linear, tree-based ensemble, and prior-fitted network—substantially strengthens confidence in this finding relative to predictors that emerged from a single model alone. This finding suggests that specific autism phenotypic characteristics, particularly those related to childhood imaginative and social play abilities, may identify adults with autism more likely to benefit from mindfulness interventions. Greater enjoyment of pretend play in childhood is associated with less severe impairments in imagination and social play domains of autism, suggesting that individuals with relatively preserved imaginative capacities may be better positioned to engage with and benefit from mindfulness practices that require mental imagery, perspective taking, and flexible attention. The broader literature on creativity and mindfulness suggests a plausible pathway: individuals with stronger imaginative or creative thinking capacities may derive more benefit from mindfulness practice. For example, a meta-analysis reported a modest positive correlation (*r*≈0.22) between mindfulness and creativity [49]. This makes sense given that many mindfulness practices involve imagination either directly (eg, guided imagery, which involves imagining a particular scene) or indirectly (eg, being asked to bring certain people to mind during an appreciation or compassion exercise) [50,51].

#### Few-Shot Learning With LLLMs

The few-shot learning experiments revealed that LLMs can effectively classify treatment responders when provided with appropriately tokenized baseline data, achieving accuracy (0.867) that substantially exceeded that of random forest (0.733) at 70 training examples, with a 13.3 percentage point improvement. This finding has practical implications for clinical prediction in specialized populations where large training datasets are difficult to obtain.

Notably, feature dimensionality critically shaped the in-context learning efficiency. The 7-feature tokenization substantially outperformed the 100-feature approach, suggesting that LLM few-shot learning does not simply scale with information richness. Rather, compact, high-signal representations appear necessary for the model’s attention mechanism to identify cross-example patterns that predict outcomes. The finding is consistent with evidence that chain-of-thought prompting benefits reasoning tasks but can hinder pattern recognition tasks where the model should learn directly from demonstrations [52]. For clinical applications, this suggests that complex information does not guarantee better performance. Because the 7 features were selected using supervised models, this experiment did not evaluate whether the LLM can identify predictive variables. It evaluated whether the LLM can use a small set of predefined clinical variables for prediction. Therefore, the observed advantage reflects classification performance given selected inputs rather than feature discovery ability.

These results should be interpreted cautiously. The few-shot approach required 70 labeled examples to achieve peak performance, which is still a meaningful data requirement for rare populations. Additionally, LLM-based predictions lack the interpretability of logistic regression coefficients or SHAP values, limiting clinical insight into why specific predictions are made. The few-shot approach is best viewed as a complementary method for settings where traditional machine learning training data is insufficient rather than as a replacement for interpretable supervised models.

### Model Performance and Methodological Considerations

The superior performance of ensemble methods (random forest and TabPFN) compared to simpler linear models aligns with the hypothesis that treatment response emerges from complex, nonlinear interactions among baseline characteristics. Random forest’s ability to capture feature interactions without explicit specification, combined with its relative resistance to overfitting through bootstrap aggregation, likely contributed to its strong performance. TabPFN’s competitive performance is particularly noteworthy given that this transformer-based foundation model requires no hyperparameter tuning and was not trained on mental health data specifically. This suggests that general-purpose tabular prediction methods may transfer effectively to mental health applications.

XGBoost’s relatively weaker performance (AUC 0.73, 95% CI 0.53‐0.92) was unexpected given its strong track record in machine learning competitions and health care applications. This may reflect the challenge of hyperparameter optimization with limited sample size (n=73), as gradient boosting methods require careful tuning to avoid overfitting. The wide CI for XGBoost suggests instability across cross-validation folds, potentially indicating that the model was fitting to noise in some folds.

### Limitations

Several limitations warrant consideration when interpreting the findings. First, the modest sample size (n=73) limits statistical power. A larger sample would enable more robust conclusions and potentially reveal additional predictive features with adequate statistical power to detect interaction effects.

The few-shot LLM analysis should be interpreted within the context of broader evidence on LLM performance in clinical classification tasks. Prior work has demonstrated that LLMs prompted without fine-tuning do not consistently outperform specialized machine learning models in neurobehavioral diagnostic tasks, even when provided with structured clinical information [53]. Our LLM results are presented as a feasibility demonstration in a low-data regime rather than a claim of general LLM superiority, and the comparison with random forest was conducted on an identical 7-feature input to ensure methodological equivalence. Nevertheless, the generalizability of our few-shot findings to other LLM architectures, tokenization strategies, or clinical prediction contexts remains uncertain and warrants investigation in future work.

The treatment response criterion (a ≥7-point STAI state score reduction), while grounded in established guidelines, represents one of several valid operationalizations. Anxiety reduction exists on a continuum, and alternative response definitions might yield different results.

The marked phenotypic diversity within autism presents inherent challenges for generalizing these predictive models. Autism is “now widely accepted as a complex, pervasive, heterogeneous condition with multiple etiologies, subtypes, and developmental trajectories,” with tremendous phenotypic heterogeneity in adaptive function, cognitive and language abilities, and neurological comorbidities, leading some researchers to refer to these various disorders as “the autisms” [54]. However, our rigorous nested cross-validation approach demonstrated moderately stable model performance across multiple held-out test sets (AUCs 0.79 for state anxiety), suggesting that the predictive patterns we identified are robust within samples similar to ours. While external validation in more diverse samples of individuals with autism remains critical to establish broader generalizability, the consistency of our findings across cross-validation folds indicates that concerns about heterogeneity, although valid, do not substantially undermine the reliability of our models for cognitively able adults with autism who can independently engage with smartphone-based interventions.

These models specifically target the Healthy Minds Program delivered via smartphone, limiting broader applicability. The generalizability of these predictive models to other mindfulness-based interventions, delivery formats (eg, in-person group therapy and web-based programs), or other types of digital mental health interventions for anxiety remains unknown.

### Future Directions

Several research directions could build upon these preliminary findings. First, external validation in an independent sample of adults with autism is essential to confirm that these predictive models generalize beyond the original study cohort. Ideally, such validation would occur in diverse samples varying in demographic characteristics, autism phenotypes, baseline anxiety severity, and geographic settings.

Second, incorporating real-time engagement data and early response indicators could enable dynamic prediction models that update as treatment progresses. For instance, models that consider adherence patterns in the first week, changes in anxiety during the first 2 weeks, or engagement with specific intervention components might achieve higher accuracy while still providing actionable predictions early enough to guide clinical decisions.

Third, research should explore the mechanisms underlying the observed predictive relationships, particularly the association between childhood pretend play and treatment response. Qualitative interviews with adults with autism who did and did not respond to mindfulness interventions could provide insights into how imaginative capacity, visualization ability, and preferred learning styles influence engagement with different mindfulness techniques. Such mechanistic understanding could inform the development of more accessible mindfulness interventions tailored to diverse cognitive styles.

Lastly, implementation research examining how clinicians, individuals with autism, and their families respond to predictive information would guide ethical and effective translation into practice. Questions include the following:

- How should predictions be communicated?
- When should predictions inform treatment selection versus treatment augmentation?
- How do predictions affect therapeutic alliance, treatment expectancies, and self-efficacy?
- What safeguards prevent inappropriate denial of treatment based on predictions?

### Conclusions

The present findings indicate that machine learning models can successfully distinguish responders from nonresponders in terms of state anxiety reduction in response to a smartphone mindfulness intervention in adults with autism. The random forest classifier achieved an AUC of approximately 0.79 in predicting STAI state anxiety responders, reflecting meaningful ability to identify individuals who would show significant anxiety reduction. Discernible baseline patterns differentiated those most likely to benefit from mindfulness-based anxiety support, with baseline state anxiety severity, age, and autism-specific characteristics (particularly childhood pretend play) emerging as key predictors across multiple modeling approaches. Predictive models performed meaningfully for state anxiety outcomes but not for trait anxiety, suggesting that acute anxiety relief is more foreseeable from baseline characteristics than broader dispositional change and underscoring the importance of distinguishing these outcomes when evaluating treatment personalization tools.

Few-shot learning with LLMs demonstrated superior performance to traditional machine learning when provided with compact, high-signal feature representations, achieving an accuracy of 0.867 at 70 training examples, compared to an accuracy of 0.733 for random forest. This approach offers a promising complementary method for clinical prediction in specialized populations where large training datasets are difficult to obtain.

To our knowledge, this is the first study to apply machine learning to predict individual clinical benefits of mindfulness training in adults with autism. While machine learning approaches have been applied to predict response to smartphone-based mindfulness interventions in other populations with anxiety [55,56], adults with autism represent a clinically distinct group for whom such personalization tools were absent. This study marks a step toward precision psychiatry in anxiety management for adults with autism. The present findings suggest that, as online interventions to support mental health become ubiquitous, it is possible for patients and clinicians to know whether a particular intervention is more or less likely to be helpful for an individual patient—a prospect now supported by a growing body of machine learning research applied to digital mental health platforms [57-59]. With further refinement and validation in larger, more diverse samples, data-driven algorithms could empower more informed decision-making and help match each adult with autism to the intervention from which they are most likely to benefit.

## Supplementary material

10.2196/89054 Multimedia Appendix 1 Large language model prompt used in few-shot learning.

10.2196/89054 Multimedia Appendix 2 Shapley Additive Explanations and calibration plots.

## Abbreviations

AQ: Autism Spectrum Quotient
AUC: area under the curve
AUC-ROC: area under the receiver operating characteristic curve
CATI: Comprehensive Autistic Trait Inventory
FFMQ-15: Five Facet Mindfulness Questionnaire-15
LLM: large language model
MAAS: Mindful Attention Awareness Scale
MBI: mindfulness-based intervention
PANAS: Positive and Negative Affect Schedule
PROMIS: Patient-Reported Outcomes Measurement Information System
PSS: Perceived Stress Scale
RCT: randomized controlled trial
SHAP: Shapley Additive Explanations
SPARK: Simons Foundation Powering Autism Research for Knowledge
SRS-2: Social Responsiveness Scale, Second Edition
STAI: State-Trait Anxiety Inventory
TabNet: tabular data network
TabPFN: Tabular Prior-Data Fitted Network
XGBoost: extreme gradient boosting
