# Supplementary material for: Large Language Model Few-Shot Learning for Predicting Individual Treatment Response to Smartphone-Based Mindfulness in Autistic Adults With Anxiety: Secondary Analysis of a Randomized Controlled Trial
Source: JMIR AI. 2026 Jul 23;5:e89054. doi: 10.2196/89054 (PMC13394852; doi:10.2196/89054)
Supplement: Multimedia Appendix 1 [file ai-v5-e89054-s001.docx]

## Multimedia Appendix 1. LLM Prompt used in Few-shot learning

### System prompt

You are a clinical evaluator who determines whether a autistic audalts patient showed a meaningful treatment response On mindfulness based intervention (Healthy Mind Program) based on natural-language descriptions of their pre-treatment state.

Use the following clinical definition of response:

A responder (label 1) is a patient whose text shows decrease of state anxiety using STAI. If the decrease was bigger than 7, it was considered as responders.

A non-responder (label 0) is a patient who shows improvement, on state anxiety that is smaller than 7

At the last, Return ONLY valid JSON: {"label": 0 or 1}.

User prompt template

Here are {N_SHOTS} examples:

INPUT: {text_1}
LABEL: {label_1}

INPUT: {text_2}
LABEL: {label_2}

...

Now classify this.

INPUT: {text}

Output format

1. Attempt JSON parsing:

If {"label": 0 or 1} → use that value

1. Regex extraction:

Pattern: "label"\s*:\s*"?([01])"?

1. Heuristic fallback:

If text ends with "1" (and not "0") → label 1

Otherwise → label 0

1. If all fail → label 0
