# Supplementary material for: Large Language Model Few-Shot Learning for Predicting Individual Treatment Response to Smartphone-Based Mindfulness in Autistic Adults With Anxiety: Secondary Analysis of a Randomized Controlled Trial
Source: JMIR AI. 2026 Jul 23;5:e89054. doi: 10.2196/89054 (PMC13394852; doi:10.2196/89054)

## Multimedia Appendix 2. Supplementary SHAP and calibration figures.

Figure S1 | SHAP beeswarm plot illustrating top-10 feature importance for the STAI state dataset using the TabNet model for state anxiety prediction.


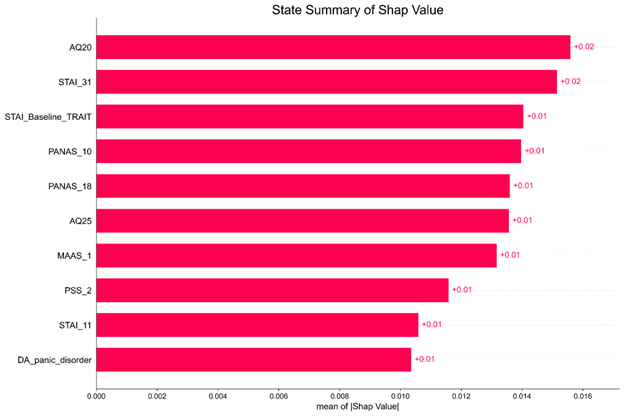


Figure S2 | SHAP value plots illustrating top-10 feature importance for the STAI trait dataset across three different models: (a) RandomForest, (b) XGBoost, (c) Tabnet and (d) TabPFN. Each plot visualizes the impact of individual features on model output, with color indicating feature value and horizontal position representing SHAP value.


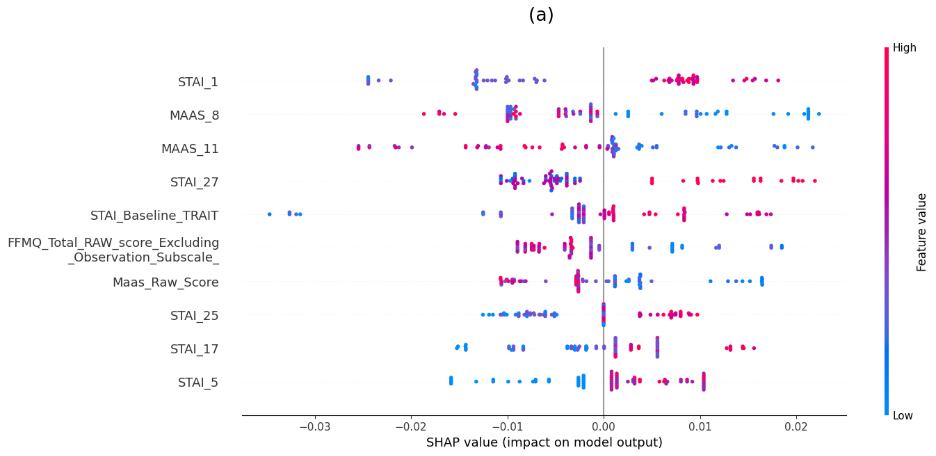


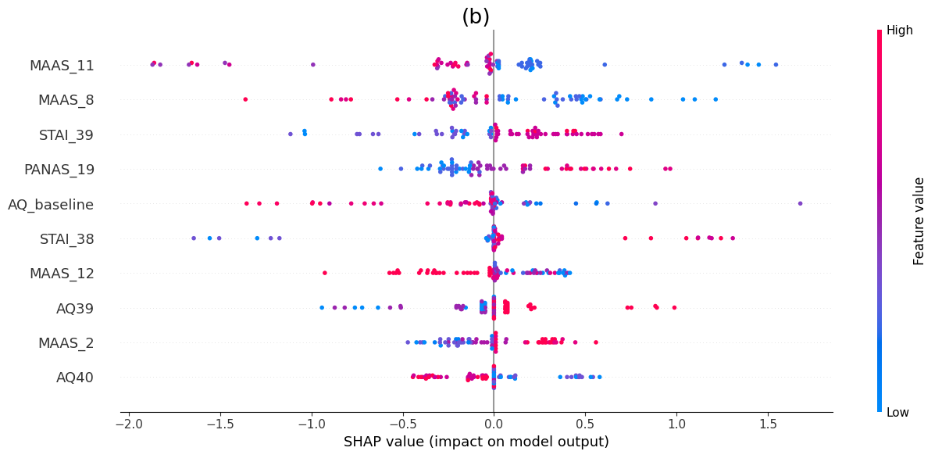


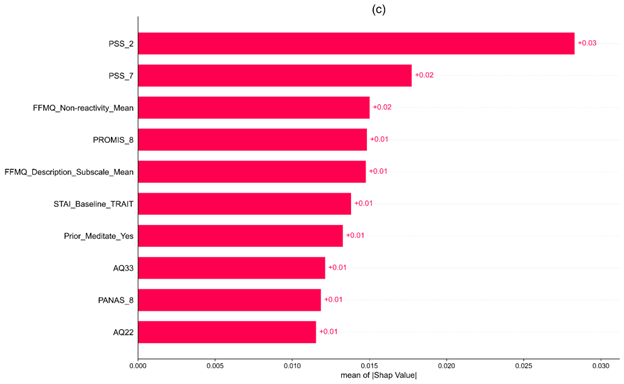


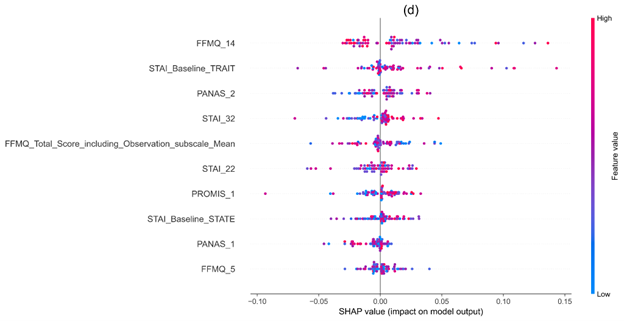


Figure S3 | Comparison of calibration plots of the aggregate 5 k-fold (stratified) cross validation for STAI state between Logistic Regression (a) and RandomForest (b). Calibration curves provide information on how well the probabilistic predictions of a binary classifier are calibrated (Sciki-learn). When the curve is above the calibration line, it indicates the model is too confident of its predictions.


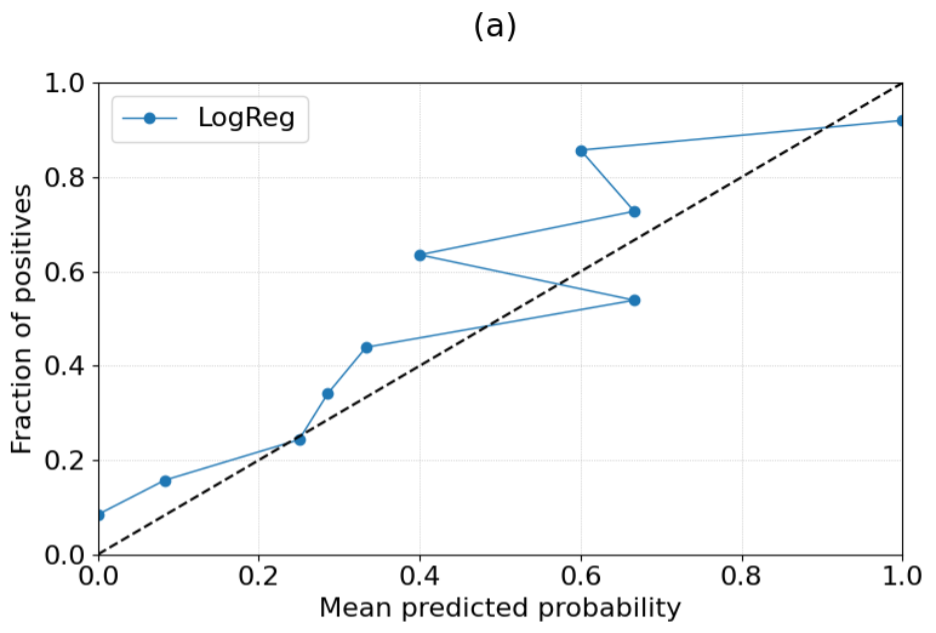


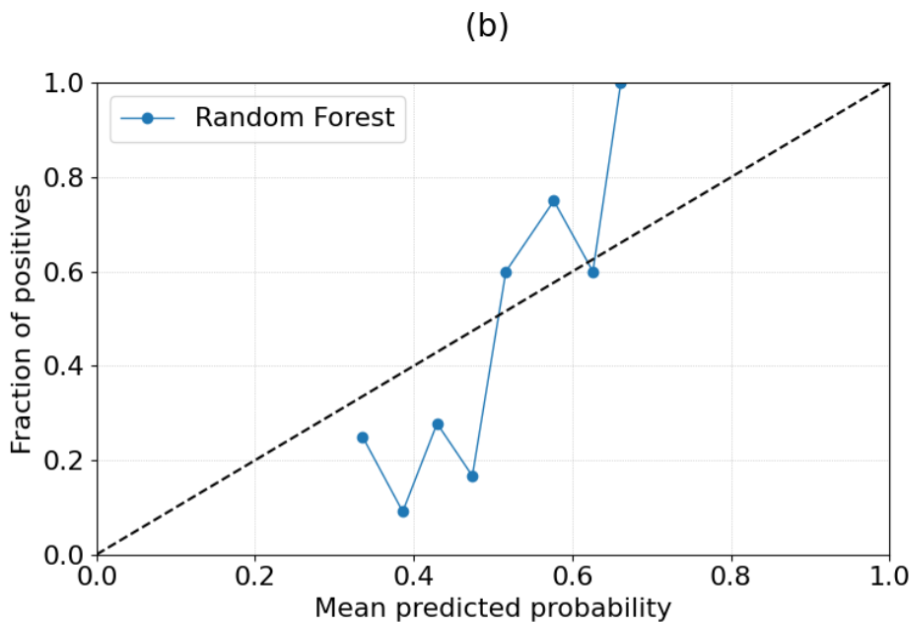

Supplement: Multimedia Appendix 2 [file ai-v5-e89054-s002.docx]
